# Mapping Moisture Sorption Through Carbohydrate Composite Glass with Fourier Transform Near-Infrared (FT-NIR) Hyperspectral Imaging

**DOI:** 10.1007/s11483-014-9376-x

**Published:** 2014-11-06

**Authors:** Christine M. Nowakowski, William R. Aimutis, Scott Helstad, Douglas L. Elmore, Allen Muroski

**Affiliations:** 1 General Mills, Inc., 330 University Ave. SE, Minneapolis, MN 55414 USA; 2 Global Food Research, Cargill, Inc., 2301 Crosby Road, Wayzata, MN 55391 USA; 3 Corn Milling North America, Cargill, Inc., Dayton, OH USA; 4 3M, Inc., Maplewood, MN USA; 5 Pepsico, Inc., Vahalla, NY USA

**Keywords:** FT-NIR spectroscopy, Sugar glass, Moisture penetration, Crystallization, Mapping, Sucrose, High fructose corn syrup, Maltotriose

## Abstract

Inherent changes in foods during storage are often caused by water sorption or desorption that often results in product matrix instability. Water sorption behavior differs depending on the matrix through which it moves. Often, concurrent phenomenon such as crystallization modifies water’s movement. We describe a novel use of hyperspectral imaging combined with Fourier Transform Near Infrared (FT-NIR) spectroscopy to map where water molecules are in two dimensions while concurrently quantifying the crystallization motif as water sorbs into a carbohydrate matrix over a month’s storage time. This methodology allows us to identify and quantify sucrose crystals formed within a carbohydrate matrix while also mapping water migration through this complex matrix. We compared corn syrup/sucrose blends where sucrose is supersaturated (high sucrose, HS), sucrose is below saturation (low sucrose, LS), sucrose below saturation with embedded sucrose crystals (LSS) and maltotriose is supersaturated within a corn syrup matrix (high maltotriose, LSM). This FT-NIR method was used to characterize water sorption through a carbohydrate matrix over time and measured both the propensity of the systems to form sucrose crystals and the influence sucrose crystals have on water sorption. We observed water diffusion was slower in lower sugar carbohydrate glasses, and the process of sorption was different. Amorphous systems supersaturated in sucrose allow crystallization when sufficient water is sorbed and thus, this concurrent action disrupts normal Fickian diffusion. The water front compresses to a narrow band as it sorbs through the matrix. The presence of embedded crystals in an amorphous matrix slows overall water penetration through the matrix by convoluting the path of moving water molecules. This did not appear to change the rate of diffusion. Experiments with maltotriose at supersaturation concentration showed the crystallization rate was slower than sucrose. Thus, pure maltotriose is not a practical solution as a potential replacement for sucrose to slow sorption in food systems.

## Introduction

The process of sorption encompasses both adsorption and absorption. Water sorption in amorphous solids is interesting to the food industry because of its impact on stability and consumer-expected product behavior [1–3]. Bulk packed products that have inadvertently sorbed additional moisture are likely to be negatively perceived by consumers as having stale off-flavor, feel ‘soggy’, or clumping in the box. Historically, glass transition is used as a concept to explain and predict observed phenomena such as stickiness of coatings or clumping as water is sorbed into a matrix [4–6]. Conversely, the lack of water sorption in products is described as crispy, crunchy and free-flowing. In amorphous solids supersaturated with respective molecules there is a tendency to crystallize; sorbed water allows for sufficient viscosity reduction for this to occur. Subsequently, another organoleptic change occurs in foods often referred to as ‘bloom’. In this case, stickiness is not necessarily a descriptor but other texture attributes, such as mushy or syrupy, could be apparent.

Water sorption rates measure average weight gain over time (sorption isotherms) to accurately describe how much total water is present in a sample with respect to time while stored at selected humidity/temperature conditions [7]. This approach has successfully shown that sorption rates differ between compositions high in sucrose content, where water sorption is influenced by concurrent sucrose crystallization, and compositions too low in sucrose for crystallization to occur [7–10]. However, these studies describe model systems as a whole and are unable to predict the impact of water on local carbohydrate hydrogen bonding as water moves through amorphous foods.

Low percentages of water (2 % or less) in a carbohydrate glass can act as a mechanical anti-plasticizer, strengthening amorphous solids [11, 12], whereas greater percentages of water reduce the rigidity (E’) of amorphous solids [13, 14]. Sucrose glasses are formed when sucrose concentration exceeds its saturation point. Sorbed water plasticize the glass so local viscosity is sufficiently lowered allowing sucrose molecules to form nuclei resulting in crystal formation [1] which is thermodynamically more stable than the glass counterpart. Additionally, viscosity reduction during this event favors kinetics for water molecules to be excluded from the crystal as it grows [15]. Free water further reduces local viscosity, thus continuing to promote favorable crystal growth dynamics [10]. However, when carbohydrate glasses are low in small molecular weight compounds, little or no crystallization is possible and thus the amorphous nature of solid is maintained as it is plasticized and begins to lower its viscosity or ‘melts’.

The presence of crystals in an amorphous matrix can impact the mechanical response of a coating [16]. Solid particles, not solubilized in the continuous amorphous structure, can act as mechanical ‘fillers’ and consequently reduce observable stickiness and/or clumping by increasing the storage modulus of the composite [16]. However, an amorphous matrix, with the inclusion of crystals, has a lowered Tg as measured by DSC due to moisture sorption as water would preferentially solubilize the carbohydrate glass.

Recent advances in spectral imaging have allowed researchers to directly image water and demonstrate how it impacts hydrogen bonding within an amorphous matrix [17].

Infrared spectroscopy is used extensively to study sugars and other carbohydrates because it is sensitive to configuration, conformation, and hydrogen bonding [18, 19]. Mid-infrared and near infrared (NIR) are well suited for providing water content and phase information simultaneously. Spatially resolved spectra are obtained on a microscopic scale using an infrared microscope. Hyperspectral imaging is possible by coupling a focal plane array (FPA detector) to a Fourier transform infrared (FTIR) microscope [20–24]. FTIR hyperspectral imaging is well suited for use in diffusion studies and obtains a large amount of spatially- and time-resolved information simultaneously [25, 26] providing extensive insight into solvent diffusion in various synthetic polymer systems.

Liang, et al. [17], demonstrated FT-NIR single point microscopy utility for studying moisture migration in sugar films. By employing a 64 × 64 MCT FPA detector, the technique is expanded to obtain high quality chemical images based on the near infrared combination band intensities. We report for the first time the methodology and results of a FT-NIR hyperspectral imaging study on moisture migration in a variety of glass phase sugar films.

## Materials and Methods

**Fig. 1:**
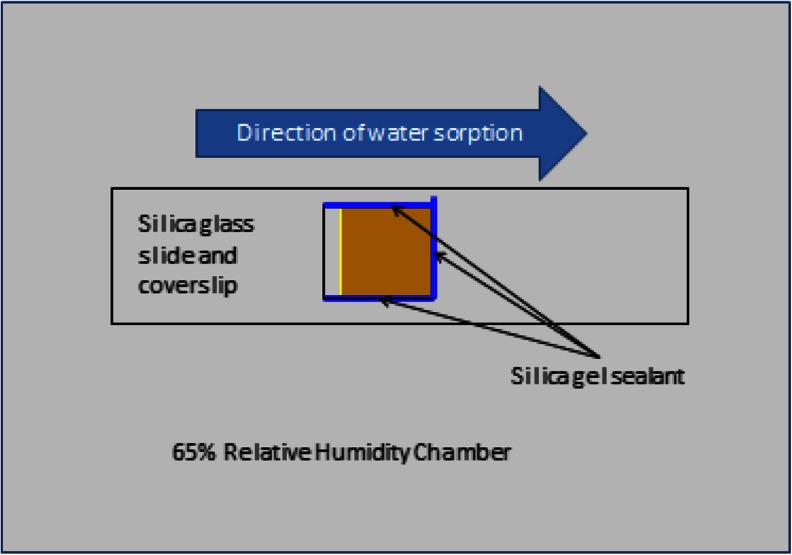
Sample preparation for incubations. In all cases the sugar film is ~1 mm thick

Carbohydrate glasses were formulated (Table 1) with ingredients from Cargill Corn Milling, Dayton, OH (36 DE corn syrup) and Sigma-Aldrich, Milwaukee, WI (sucrose, maltotriose). Carbohydrate glasses were prepared by heating to 114+/− 2 °C and placing one drop of molten syrup onto a pre-heated (125 +/− 2 °C) silica glass microscope slide placed on a hot plate (VWR International, Radnor, PA). Sample was held on the heated surface approximately 5 min, until melt moisture reached 2 +/− 0.5 % as measured by Karl Fischer. A cover glass was then placed on top of the melt, and sample was removed to cool to room temperature. For the sample that required added sucrose crystals, these were evenly distributed over the melt just prior to covering with the glass plate and cooling. After samples were cooled, they were transferred to a desiccator over dry calcium sulfate. Samples were removed from desiccation as needed, sealed on three sides by silica gel, reinforced with tape (Fig. 1), and placed in a 65 +/−5 % relative humidity chamber over cupric chloride saturated salt solution. Samples were incubated for up to 4 weeks at room temperature. Samples were removed briefly as needed for spectral analysis.

**Table 1:** Formulations of melts used in this study

	High sucrose (HS)	Low sucrose – corn syrup (LS)	Low sucrose –embedded sucrose crystals (LSS)	Low sucrose – maltotriose (LSM)
Sucrose	70	63	55	63
Corn syrup 63 DE	7	0	0	0
Corn syrup 36 DE	0	20	20	0
Sucrose crystals	0	0	2	0
High fructose corn syrup	8	8	8	8
Maltotriose	0	0	0	14
Water	15	9	15	15
Total	100	100	100	100

### FT-NIR Hyperspectral Imaging

FT-NIR hyperspectral images were acquired using a Varian/Agilent UMA 600 FTIR microscope, coupled to a Varian/Agilent FTS 7000 rapid-scan spectrometer. Varian’s Resolutions Pro 4.1 software managed instrument control and data acquisition. The microscope was equipped with a 64 × 64 mercury cadmium telluride (MCT) focal plane array detector (Santa Barbara Focal Plane/Lockheed Martin). Sample illumination was via the FTS-7000’s tungsten-halogen light source and interferometer, fitted with a CaF_2_ beam splitter. We collected images with the UMA 600 in transmission mode, using 15x Cassegrain objective and condenser elements, focusing at the center of the sample depth. The field-of-view in this case is 350 × 350 μm. Spectral data within the image are the result of co-adding 100 interferograms at 8 cm-1 spectral resolution over the range 6,000 to 4,000 cm^−1^, followed by Fourier transformation with a triangular apodization function using an under-sampling ratio of two. These single-beam data were then ratioed to a background image collected, using the same spectral parameters, through a single blank glass slide. We employed 2 × 2 binning of the FPA’s 4,096 detector elements during image acquisition; therefore, each binned pixel views an 11 × 11 μm sample area. We used an automated x-y stage (Prior Scientific, Rockland, MA) with the stage control functionality of Resolutions Pro to assemble a 10 × 2 array of individual 350 × 350 μm images, producing image mosaics covering 3.5 × 0.7 mm of each sample. Each of these mosaic hyperspectral images contains 20,480 (320 × 64) pixels, with a FT-NIR spectrum associated with each of these pixels. Total acquisition time for each mosaic was about 40 min.

### Spectral Processing

Spectral manipulation was performed using Varian Resolutions Pro 4.1 software, followed by image rendering via Microsoft Excel 2002. Contrast within the percent water maps was produced via a color scale representing the range of normalized water absorption band areas across the hyperspectral image. We defined this water band over the range 5,290 to 5,010 cm^−1^, with a single-point baseline at 5,400 cm^−1^. We normalized the water band to the area between 4,975 and 4,200 cm^−1^, base-lined also at 5,400 cm^−1^. Calibration of this band region was correlated to water content determination by Karl Fischer.

Contrast within the phase maps was derived from pixel-to-pixel variation in the center-of-mass of the carbohydrate absorption band, defined between 4,960 and 4,480 cm^−1^, using a two-point baseline correction: 4,480 cm^−1^ and the extreme between 5,100 and 4,900 cm^−1^.

### Light Microscopy

Transmitted light images of the sugar films were collected using an Olympus BX-61 compound microscope, equipped with a 20x, 0.50 NA objective (UPlan FL N), and a tungsten-halogen light source. Image capture was via an Olympus DP72 color cooled CCD camera, at 2,048 × 1,536 pixels. Polarized light images were captured with this same configuration using the Olympus U-PO accessories. Macroscopic transmitted-light images of the sugar films were collected using an Olympus SZX-16 stereoscope, using a 1X, 0.15NA objective (SDFPLAPO1 x PF), and an Olympus DP71 cooled color CCD camera, at 2,048 × 1,536 pixels.

## Results and Discussion

Water sorption proceeded from a high moisture environment (65 % relative humidity) towards a low moisture environment (2 % moisture carbohydrate matrix) in amorphous films regardless of carbohydrate composition. The rate and path of moisture movement is formula-dependent. These observations are in agreement with Liang, et al. [17]. We expand upon that work and employ a novel method for more detailed understanding of water molecular movement while simultaneously measuring the impact on crystallinity.

This new method allows simultaneous visualization of spatial differences in water content and carbohydrate phase in sugar films. Overlaid %water and phase profiles allowed detailed correlations between %water, phase and distance. Additionally, spectroscopic images reveal the two-dimensional nature of the water front, and heterogeneity in terms of crystallinity and water content. The images also show the size, shape, and location of crystalline islands and voids (bubbles), and it is also possible to monitor density in similar manner.

### Image construction

**Fig. 2:**
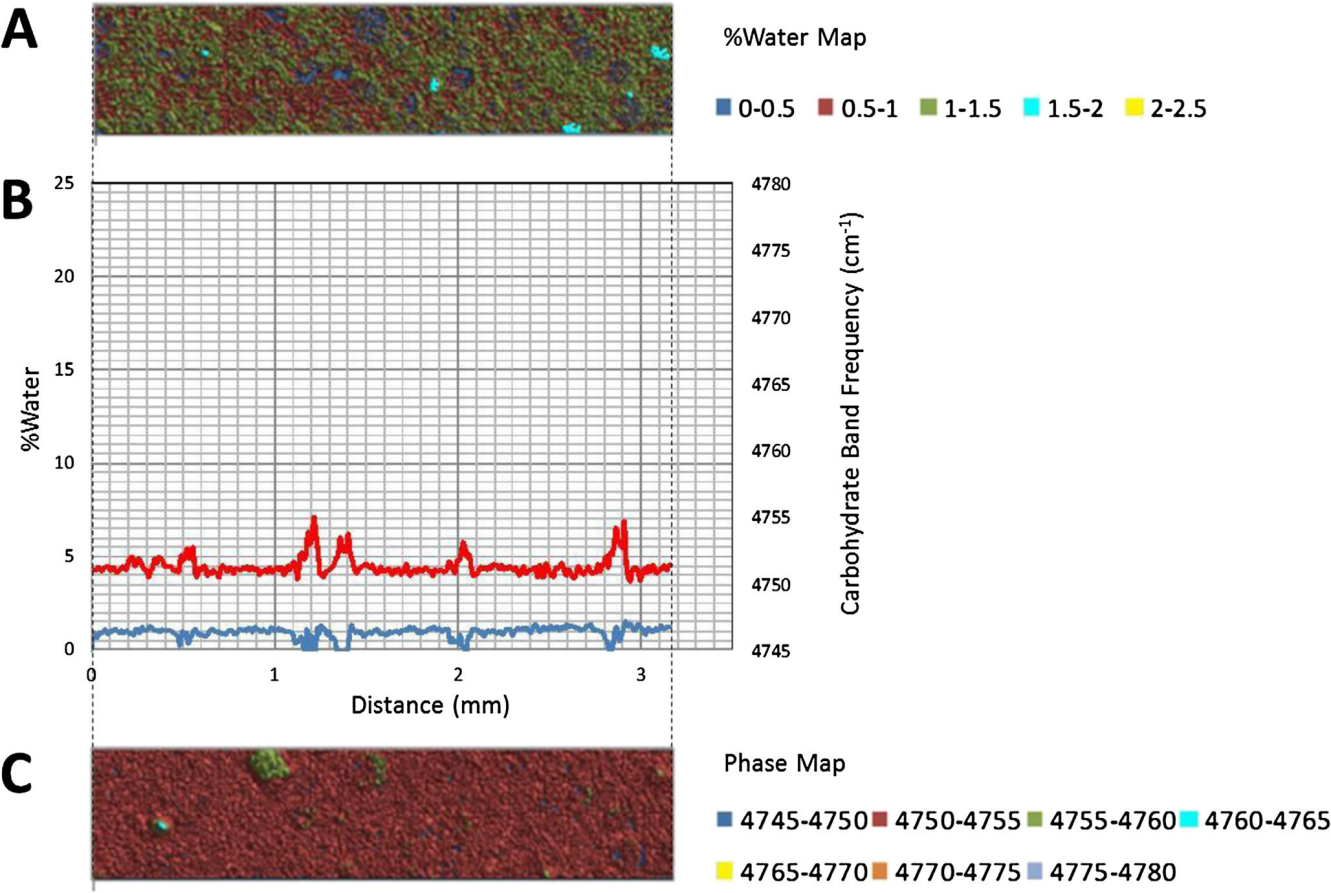
%Water map (**a**), Overlaid %water and phase profiles (**b**), and phase map (**c**) for high sucrose (HS) at 0 weeks

**Fig. 3:**
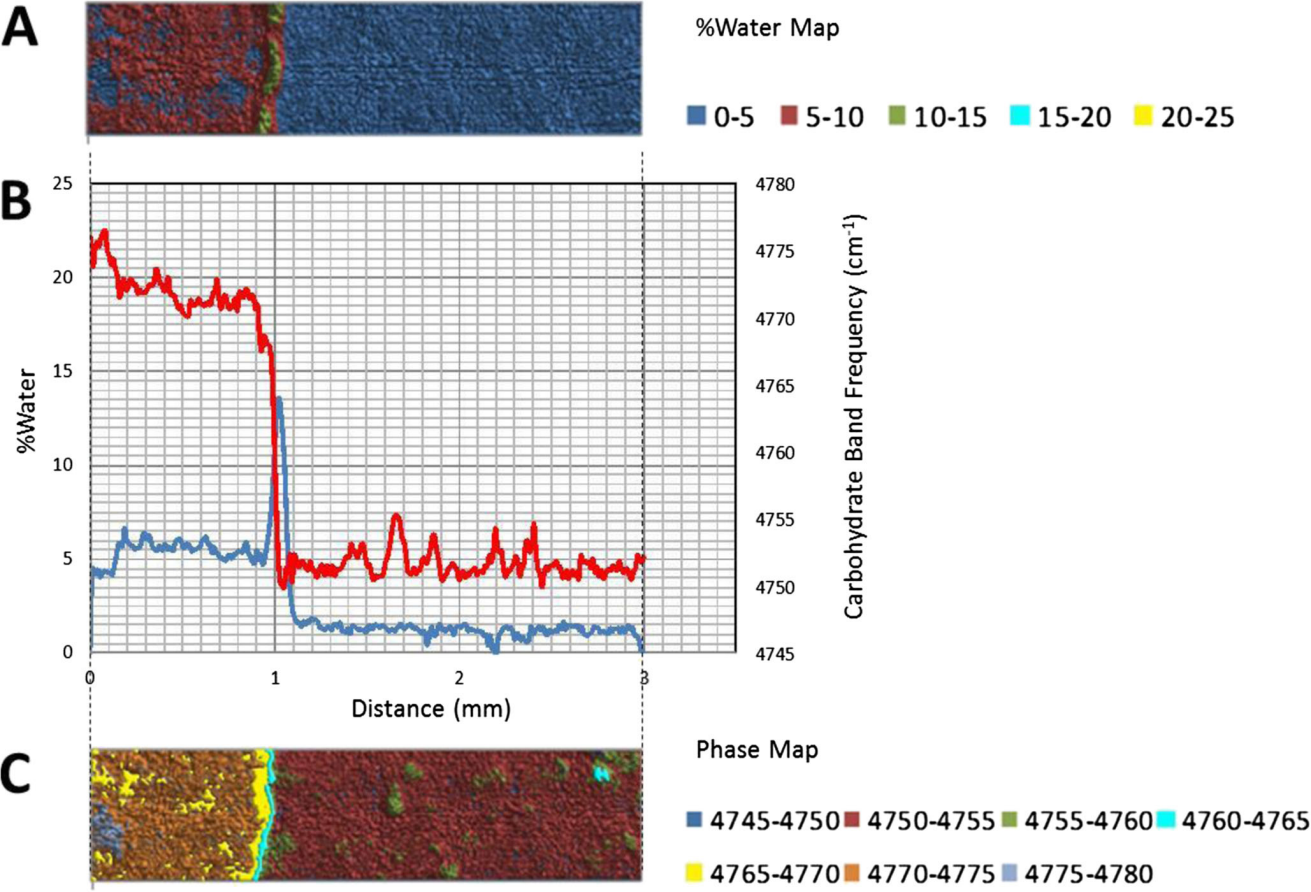
%Water map (**a**), Overlaid %water and phase profiles (**b**), and phase map (**c**) for high sucrose (HS) at 2 weeks

**Fig. 4:**
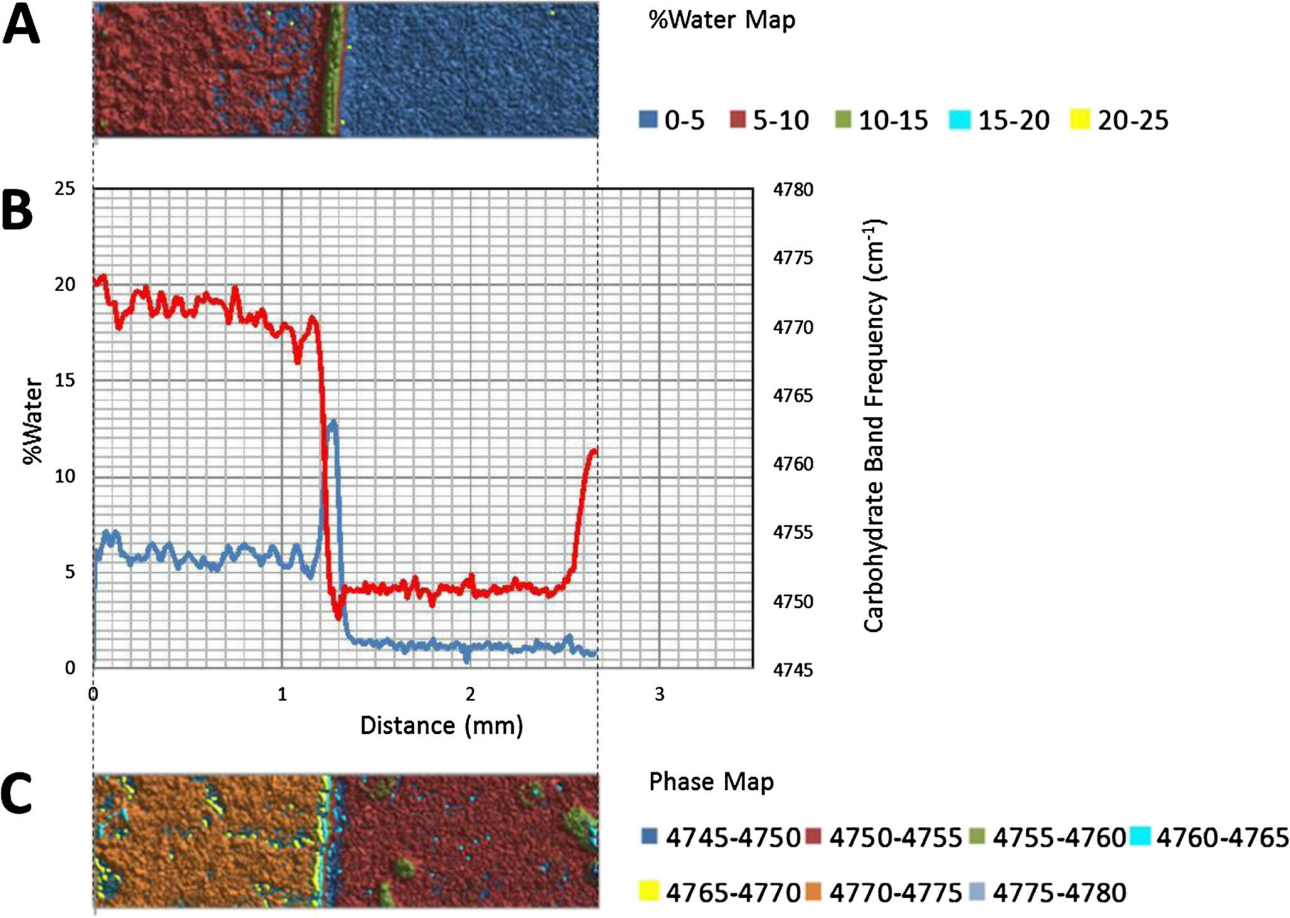
%Water map (**a**), Overlaid %water and phase profiles (**b**), and phase map (**c**) for high sucrose (HS) at 4 weeks

**Fig. 5:**
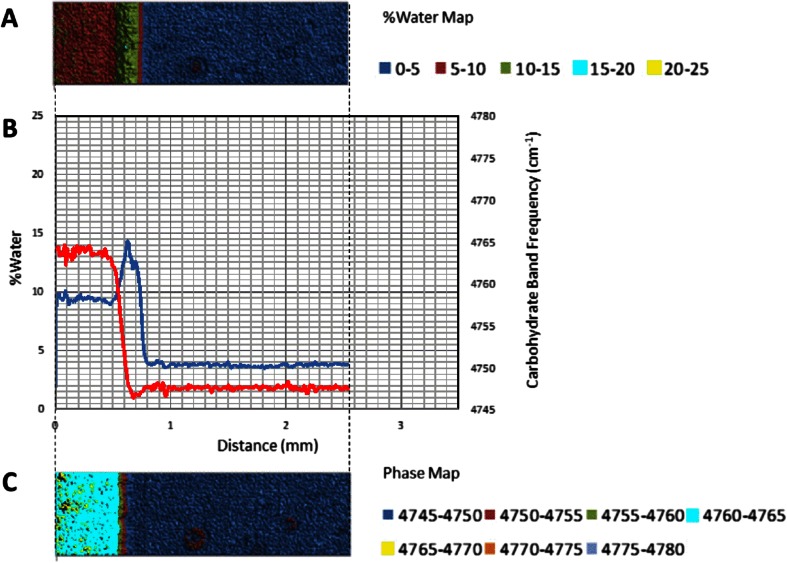
%Water map (**a**), Overlaid %water and phase profiles (**b**), and phase map (**c**) for low sucrose – maltotriose (LSM) at 4 weeks

**Fig. 6:**
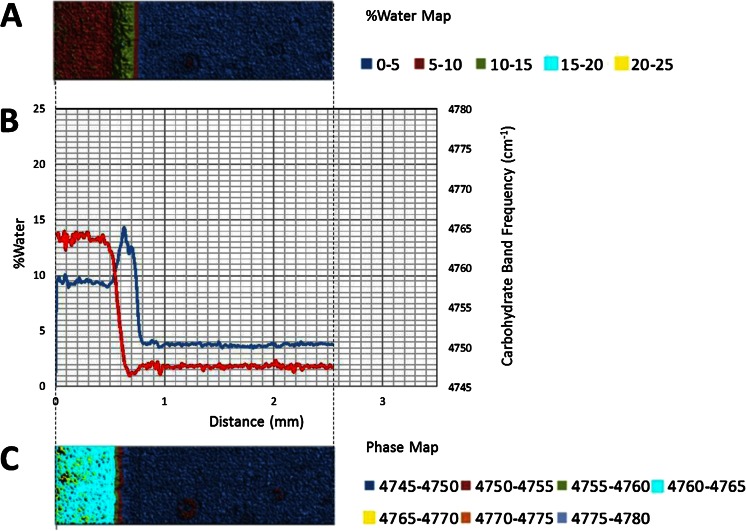
%Water map (**a**), Overlaid %water and phase profiles (**b**), and phase map (**c**) for low sucrose – corn syrup (LS) at 4 weeks

**Fig. 7:**
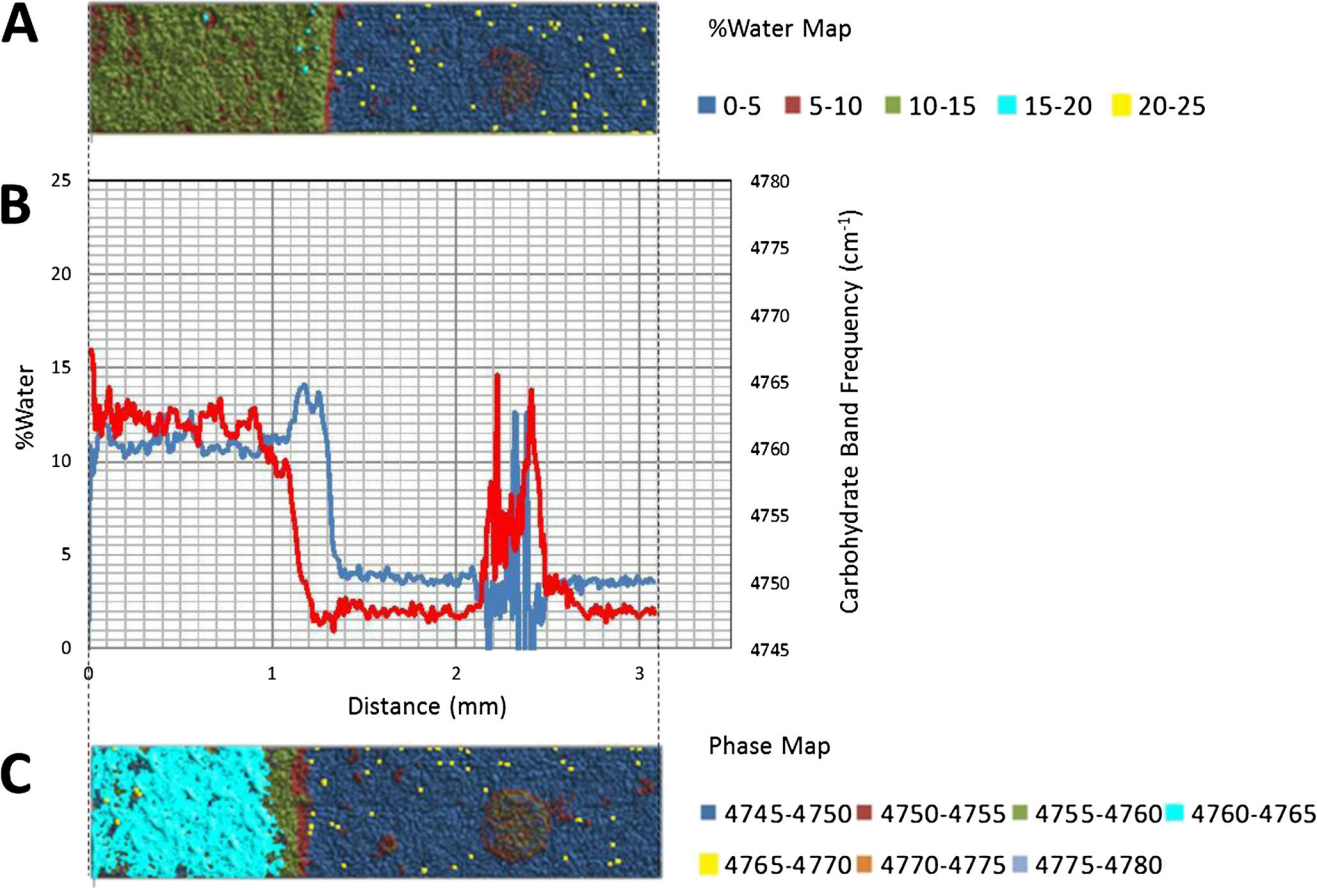
%Water map (**a**), Overlaid %water and phase profiles (**b**), and phase map (**c**) for low sucrose corn syrup with embedded sucrose crystals (LSS) at 4 weeks

The 5,150 cm^−1^ band is assigned to a combination of fundamental stretching and bending vibrations in water, while the 4,800 cm^−1^ band is assigned to a combination of fundamental stretching and bending vibrations in sugar molecules. The bands were chosen for this study because they are well resolved, provide optimal absorbance values in a ~1 mm sample and can be monitored with a conventional MCT based FTIR microscope fitted for FT-NIR measurements. A legend correlating %water values and color is provided below the map. Overlaid plots of %water and 5,150 cm^−1^ sugar band frequency values (line profiles) as a function of distance show water migration along the sugar film (Figs. 2, 3, 4, 5, 6 and 7). One film edge is designated as 0 mm is exposed to 65 % relative humidity. The line profiles correspond to a 77 μm thick line running through the center of the sugar film parallel to the horizontal axis of glass slide (Fig. 1). The frequency of 4,800 cm-1 band reveals changes in the phase of sugar films, i.e. rubbery, glass or crystalline [27]. Phase maps created using 4,800 cm-1 carbohydrate band frequencies are in Figs. 2a, 3a, 4a, 5a, 6a and 7a. A legend correlating frequency water values and color is below the map.

Quantitative changes in water content along the horizontal axis of film are observed in the %water line profiles (Figs. 2a, 3a, 4a, 5a, 6a and 7a). The phase line profiles are overlaid to show the relationship between water content and carbohydrate phase. Three distinct regions are apparent in the %water maps including a low water region associated with amorphous solid (glass) phase sugar/syrup, a high water peak-shaped moisture front and a medium water content region after the moisture front (Figs. 2b, 3b, 4b, 5b, 6b and 7b). Phase maps (Figs. 2c, 3c, 4c, 5c, 6c and 7c) show this medium region is partially crystalline (and somewhat heterogeneous).

### Chemical Images and Line Profiles

#### High Sucrose (HS)

Spectroscopic results for the HS formula are presented for 0, 2, and 4 weeks incubation, respectively (Figs. 2a–c, 3a–c and 4a–c). In addition, chemical maps and line profiles for each incubation period are shown. . In the plots and maps water migrates through the sugar films from left to right. However it is convenient to describe the effects of water migration from right to left, as the portion of the film that most recently interacted with migrating water is on the right. The plots and maps can be thought of as “snap shots” of the kinetics process, where process begins at the unaffected portion of the film just to the right of the migrating water front. From left to right we begin by describing the initial state of the film and end with the most recent of the state. For each case the maps and plot are presented with a common distance axis to facilitate interpretation.

At 0 weeks incubation, sucrose-syrup glass exists as originally cast on a slide and has not been exposed to high humidity (Fig. 2a–c). The maps and profiles reveal a continuous amorphous, low moisture (approx. 1 % water) region with small sucrose crystalline domains dispersed throughout (Figs. 2a–c). After 2 weeks incubation, maps and profiles show an area of higher water concentration moving progressively inward per incubation time (Figs. 3a–c). The beginning of high water concentration region moves from 0 to 1.1 mm and 1.35 mm at 0, 2, and 4 weeks’ time, respectively (Figs. 2a–c, 3a–c, and 4a–c). After 2 weeks FTIR indicated a maximum moisture content of 12.5 % at the glass interface. This value reduces to 5–7 % moisture just behind the higher moisture region. The movement of water suggests an opposing force to water sorption. These observations agree with Nowakowski and Hartel [7], who observed non-Fickian moisture sorption in supersaturated sucrose/syrup glasses. They attributed their observation of a concentrated moisture band to the influence of an opposing force to sorption due to high viscosity of the glassy matrix. The complimentary data of sucrose crystal concentration of the same glasses over the same time period, shows sucrose converts from amorphous glass to crystal as water sorbs through the glass, resulting in a concentration of sucrose at 0.9 mm just behind the water front, and continuing beyond back to the glass origin at 0 mm. As matrix viscosity decreases due to sorption of added water and the glass transition temperature approaches environmental conditions, the supersaturated sucrose solution has sufficient propensity to crystallize. Crystallization also contributes to forces influencing distribution of water molecules in the matrix. Thus, it appears a combination of factors including the opposing force of high viscosity in the lower moisture glass portion, reduced viscosity of the melt, and exclusion of water molecules within the sucrose crystal motif contribute to water accumulating at the interface to form a narrow region of very high water concentration. Sucrose crystallization was previously shown to force water away from the crystal front [10, 15].

Other observations are made between 0 and 2 mm at time 0 (Fig. 3a). From the %water profile, the glass phase existing between 1.1 and 2.0 mm has a water content of ~1 %. Variations were observed in the glass phase, from measurement noise or alternatively it indicates glassy regions are not homogeneous when observed with this level of precision. The standard deviation of the %water values between 1.1 and 1.7 mm is 0.15 %., indicating a variation of <±0.2 %.

At ~1.15 mm, a sharp water front peak forms (Fig. 3a). The overlaid phase profile reveals at 1.1 mm the beginning of the glass-rubbery phase transition at ~4 % water. Specifically, the water level continues increasing to a maximum of ~13.5 % water at 1.05 mm. After this point the moisture level decreases rapidly to a minimum of ~5 % water at 0.9 mm. The overlaid water and phase profiles indicate the decreasing moisture level occurs as the result of a rubbery-crystalline transition. A water level of ~5 % is observed throughout the crystalline region (0.9 mm to 0.0 mm). In this region variations in crystallinity levels and %water are anti-correlated (changes occur simultaneously in opposite directions). The variations reflect heterogeneity, not variation in spectral measurements (changes in %water are >0.1 %). The two-dimensional heterogeneity of the crystalline phase is apparent in the %water and phase maps as the glasses age (Fig. 3b and c). Furthermore, the phase image reveals presence of crystalline islands in the glass phase (1.1 to 2.0 mm), reflecting the unstable nature of the glass phase containing supersaturated sucrose levels.

Comparison of the 2 and 4 weeks samples reveals similar changes induced by moisture migration (Figs. 3 and 4). The maximum moisture in both the two and 4 weeks samples is 13.5 and 13.0 %, respectively (Figs. 3a and 4a). However, water migrates further in the 4 weeks sample. The moisture front peak maxima are observed at ~1.05 mm after 2 weeks and ~1.3 mm after 4 weeks samples (Figs. 3 and 4). The values reflect the expected exponential decrease in moisture migration rate with time, and highlight this analytical technique can be used in future studies to quantitatively determine moisture migration kinetics. A heterogeneous crystalline phase is also observed in the 4 weeks sample, with an average level of ~5.5 % water, slightly higher than the 2 weeks sample. Anti-correlation between %water and crystallinity level is observed for both samples in the crystalline phase. Crystalline islands are observed in the glass phase of both samples (Figs. 3c and 4c). The islands appear larger in the 4 weeks sample, but unfortunately the image represents different fields of view. While monitoring crystal growth in two-dimensions was not a goal in this study, this technique has potential for that purpose.

#### Low Sucrose - Maltotriose (LSM)

A set of chemical maps and line profiles for a LSM 4 weeks sample are presented in Fig. 5. Like the HS sample, three distinct regions are apparent in the %water map: a low water/glass phase region, a high water peak shaped moisture front, and a medium water crystalline region after the moisture front. From %water profile, we see the glass phase that exists between 0.8 and 2 mm has a water content of ~4 %. This value is higher than HS (~1 %). At 0.8 mm a sharp peak shaped water front begins to form. Specifically, the water level begins increasing at 0.8 mm and continues to a maximum of 14.5 % water at ~0.65 mm, then decreases to a minimum of ~9.5 % water at ~5.0 mm (Fig. 5a). The overlaid phase profile reveals the beginning of a glass-rubbery phase transition at ~0.8 mm (at ~4 % water) followed by a rubbery-crystalline phase transition at 0.7 mm. Water migrates through the LSM sample at slower rate than it does through the HS sample (1.35 mm in 4 weeks). The water front is ~0.3 mm wide (beginning to end), ~50 % wider than the water front in the HS 4 weeks sample. Moisture migrates ~50 % further in the 4 weeks HS sample compared to the LSM sample. In this specific case, the moisture front width is therefore a function of the overall moisture migration rate. This is not entirely unexpected as in relaxing melts, viscosity is the predominant opposing force to diffusion. Furthermore, a constant mass addition (rather than constant molar addition), low molecular weight additives (e.g. sucrose) would have lower viscosity than high molecular weight additives.

A crystalline region is observed between ~0.0 and 0.5 mm region for LSM 4 weeks sample (Fig. 5a). The region contains ~9.5 % water, a much higher level than in the HS sample (~5.5 %). This region is more homogeneous than the corresponding region in the HS 4 weeks sample. Furthermore fewer crystalline islands are observed in the glass phase of LSM 4 weeks (Fig. 5c). It seems plausible a slower moisture migration, and their slow crystal formation, is conducive to more homogeneous crystalline sucrose films.

#### Low Sucrose- Corn Syrup (LS)

Interestingly, some properties of the LS 4 weeks sample are similar to the HS sample, while others are similar to LSM sample (Fig. 6a). A low water, glass phase region, a high water peak shaped moisture front and a medium water crystalline region after the moisture front are observed in the %water map (like both the HS and LSM sample) (Fig. 6a). From the %water profile, the glass phase that exists between 2.0 and 1.4 mm has a water content of ~4 % similar to the LSM 4 weeks sample. The glass-rubbery phase transition occurs near 1.4 mm at ~4.5 % water. The width of the peak shaped water front appears to be ~0.3 mm (like the LSM sample). The rubbery-crystalline phase transition occurs near 1.2 mm at ~14.0 % water. The water front maxima in all samples (including the LSS sample), are all 13–14 %. Furthermore all samples began the glass-rubbery phase transition at ~4 % water (Figs. 2a, 3a, 4a, 5a and 6a). The HS sample provides useful information about this transition, since the HS sample’s glass phase contains only ~1 % water. Water profiles for the HS samples show the glass-rubbery transition begin at ~4 % water. We therefore conclude all four samples experience a rubbery-glass phase transition between 4 and 14 % as moisture migrates through the glass phase. We also conclude if a rubbery-crystalline phase transition is possible, it will occur at ~14 %. A water level of ~11.0 % is observed in crystalline region of the LS 4 weeks sample from 1.1 to 0.0 mm (like the LSM sample); in this region variation in crystallinity level and %water are anti-correlated (like the HS sample).

In contrast to the LSM 4 weeks sample, the %water and phase maps for LS (Fig. 6b and c) reveal a relatively heterogeneous crystalline phase (similar to the HS sample). The phase map (Fig. 6c) also reveals the presence of small crystalline islands in the glass phase (1.4 to 2.0 mm).

#### Low Sucrose - Embedded Sucrose Crystals (LSS)

Addition of sucrose crystals into a low sugar corn syrup glass manages water differently than when the same amount of sucrose is part of the amorphous matrix (HS) and when sucrose crystals are not present (LS) (Fig. 7a–c). In contrast to all other samples, peak moisture is not uniform at a specific distance through the matrix over time, rather what is observed there is a deviation in the % water peak due to a sucrose crystal. From 0 mm to approximately 0.7 mm, the water concentration is higher and crystallinity is decreased. From 0.7 to 1.0 mm % water is still elevated, similar to LS. At 1.0 to 1.25 mm, the glass to crystalline transition is difficult to quantify as the crystalline island disrupts flow. From 1.25 to 2 mm, crystal distribution is heterogeneous and constant (Fig. 7c). The images outline expected shapes of un-melted sucrose crystals as added to the amorphous matrix in this region. Overall, water migration is not as far as in other samples (HS & LS, respectively) indicating that by disassociating sucrose from the melt, the path water takes to sorb into a glass can be strongly influenced by crystal formation. The practical outcome of this observation is that the mechanical strength of a glass can be influenced by a change in sucrose addition.

## Conclusions

This paper describes using FT-NIR hyperspectral imaging methodology to accurately image water content and carbohydrate phases simultaneously. This method enables two dimensional mapping of water as it moves through an amorphous carbohydrate solid with ~1 mm thickness, while concurrently imaging state changes from amorphous to crystalline sucrose. Practical applications of this method will allow better understanding of water sorption and enable correlation to other physical phenomenon such as stickiness. The method will also allow more detailed modeling beyond currently described WLF or Arrhenius kinetics. In this particular example, water moves through glasses differently depending on the propensity for a substance to crystallize. The coordinated impact of both water sorption and crystallization on mechanical behavior can now be seen rather than just described via theory or endpoint observation (e.g., product clumping).
